# Magnetic Nanoparticle-Mediated Orientation of Collagen Hydrogels for Engineering of Tendon-Mimetic Constructs

**DOI:** 10.3389/fbioe.2022.797437

**Published:** 2022-03-17

**Authors:** Abigail L. Wright, Lucrezia Righelli, T. J. Broomhall, Hannah C. Lamont, Alicia J. El Haj

**Affiliations:** Healthcare Technologies Institute, Department of Chemical Engineering, University of Birmingham, Birmingham, United Kingdom

**Keywords:** collagen, tendons, magnets, alignment, hydrogels, tissue engineering, biomaterials

## Abstract

Despite the high incidence of tendon injuries worldwide, an optimal treatment strategy has yet to be defined. A key challenge for tendon repair is the alignment of the repaired matrix into orientations which provide maximal mechanical strength. Using oriented implants for tissue growth combined with either exogenous or endogenous stem cells may provide a solution. Previous research has shown how oriented fiber-like structures within 3D scaffolds can provide a framework for organized extracellular matrix deposition. In this article, we present our data on the remote magnetic alignment of collagen hydrogels which facilitates long-term collagen orientation. Magnetic nanoparticles (MNPs) at varying concentrations can be contained within collagen hydrogels. Our data show how, in response to the magnetic field lines, MNPs align and form string-like structures orientating at 90 degrees from the applied magnetic field from our device. This can be visualized by light and fluorescence microscopy, and it persists for 21 days post-application of the magnetic field. Confocal microscopy demonstrates the anisotropic macroscale structure of MNP-laden collagen gels subjected to a magnetic field, compared to gels without MNP dosing. Matrix fibrillation was compared between non- and biofunctionalized MNP hydrogels, and different gels dosed with varying MNP concentrations. Human adipose stem cells (hASCs) seeded within the magnetically aligned gels were observed to align in parallel to MNP and collagen orientation 7 days post-application of the magnetic field. hASCs seeded in isotropic gels were randomly organized. Tenocyte-likeness of the cells 7 days post-seeding in collagen I scaffolds was confirmed by the positive expression of tenomodulin and scleraxis proteins. To summarize, we have developed a convenient, non-invasive protocol to control the collagen I hydrogel architecture. Through the presence or absence of MNP dosing and a magnetic field, collagen can be remotely aligned or randomly organized, respectively, *in situ*. Tendon-like cells were observed to organize in parallel to unidirectionally aligned collagen fibers and polydirectionally in non-aligned collagen constructs. In this way, we were able to engineer the constructs emulating a physiologically and pathologically relevant tendon niche. This can be considered as an innovative approach particularly useful in tissue engineering or organ-on-a-chip applications for remotely controlling collagen matrix organization to recapitulate the native tendon.

## Introduction

Despite the high frequency of tendon injuries and their associated morbidity (Lemme et al., 2018; Nichols et al., 2019), the optimal treatment strategy remains controversial (Kadakia et al., 2017; Park et al., 2017). Regenerative approaches are a promising alternative to current surgical repair, which is prone to re-rupture and other complications, failing to address the clinical need. One such example is stem cell transplantation at the lesion site delivered within biomimetic and biocompatible constructs. This has been shown to improve the healing response and decrease the scar formation following tendon injury (Sahni et al., 2014; Yee Lui, 2015). Promisingly, it has been reported that stem cell fate can be modulated and controlled through topographical cues within its microenvironment (Shi et al., 2017; Zhang et al., 2018). Other possibilities include bioengineering of tissue analogous to the native tendon tissue for engraftment or *in vitro/in silico* modeling for fundamental research and drug screening (Lim et al., 2019).

Recapitulating the organization of the hierarchical native architecture presented within the tendon niche is essential to the success of regenerative approaches within this field. Owing to its biocompatibility and biodegradability, type I collagen is a primary component of the native extracellular matrix (ECM), making the protein an ideal biomaterial for regenerative therapeutics, specifically in the areas of tissue engineering and drug/stem cell delivery (Lomas et al., 2013; Zeltz and Gullberg, 2016; Sarrigiannidis et al., 2021). However, achieving refined modulation of the ECM architecture *ex vivo* and *in situ* has proved to be challenging. Fabrication of electrospun fibers to mimic such architecture (Beldjilali-Labro et al., 2018) or the application of 3D printing to achieve cell and ECM alignment (Khorshidi et al., 2016; Jun et al., 2018) has offered solutions. Nevertheless, the main limitation to these current methods is the inability to achieve and modulate ECM topography *in situ*.

In this work, we exploit the biological properties of collagen type I and the magnetic potential of biocompatible iron oxide magnetic nanoparticles (MNPs) to remotely control collagen hydrogel topography using a magnetic field (Weissleder et al., 1989; Berry, 2005; Hofmann-Amtenbrink et al., 2010; Jiang et al., 2019; Matos et al., 2020). We demonstrate engineering collagen gel fibrillar-like topography through the application of the magnetic field to MNP-dosed collagen hydrogels. Using MNPs, we can facilitate segmented collagen I hydrogels with fiber-like structures organized in parallel with MNP orientation. Such hydrogels demonstrate anisotropy. Human adipose stem cells (hASCs) align in parallel with fiber-like structures. In summary, we have developed a non-invasive protocol for the remote controlling collagen I matrix and cell alignment *in vitro* with potential for regenerative therapeutic applications.

## Materials and Methods

### MNP Activation and Labeling

As previously described (Rotherham and Haj, 2015; Gonçalves et al., 2018; Markides et al., 2018), dextran-coated, carboxyl-functionalized 250-nm MNPs (nanomag ®-D, 09-02-252, Micromod) were activated and, when appropriate, labeled with anti-collagen I antibody (Abcam PLC, ab260043)—hereafter “anti-collagen MNPs”—by carbodiimide activation. Briefly, 1 ml of 1 mg/ml MNP stock dispersion was prepared by dissolving 12 mg of N-(3-dimethylaminopropyl)-N′-ethylcarbodiimide hydrochloride (EDAC) (Life Technologies, E2247) and 24 mg of N-hydroxysuccinimide (NHS) (Sigma, 130672) in 2 ml of 0.5 M 2-(N-morpholino)ethanesulfonic acid (MES buffer) (Sigma, m3671) in water (adjusted to pH = 6.3 with 2M Na_2_CO_3_). A total of 20 µl was withdrawn and homogenized with 0.1 ml of MNP stock solution (10 mg/ml). The solution was mixed continuously for 1 h at room temperature (RT). Afterward, MNPs were washed in 0.2 ml of 0.1 M MES buffer in water (adjusted to pH = 6.3 with 2 M Na_2_CO_3_ in water) in a magnetic field generated by a permanent magnet. This washing step was repeated two more times with 0.1 ml of 0.1 M MES buffer. MNPs were resuspended in 0.1 ml of 0.1 M MES buffer, and 20 µl of secondary antibody (Abcam PLC, ab97048) was added. The MNPs were incubated at 4°C overnight or at RT for 3 h under continuous mixing. After the incubation time, the MNPs were washed and resuspended with 0.1 ml of 0.1 M MES buffer. The MNPs were incubated at 4°C overnight or at RT for 3 h with anti-collagen I antibody, considering 10 µg is required to saturate 1 mg of MNPs. After the incubation time, 10 µl of 25 mM glycine (Sigma, G8898) in PBS was homogenized in the MNP suspension and mixed continuously for 30 min at RT. Finally, MNPs were washed with 0.1 ml 0.1% BSA (Cells Signaling Technologies, 9998S) in PBS and resuspended in 1 ml 0.1% BSA.

### Fluorescent Collagen Labeling

To fluorescently tag collagen gels, the method by was modified (Doyle, 2018). Briefly, 5 ml of 3 mg/ml type I rat-tail collagen solution (Corning, 354249) was polymerized at room temperature for 30 min. Thereafter, collagen gels were incubated with 50 mM borate buffer solution (Fisher Bioreagent, BP168-500) (pH 9.0) for 15 min at RT. The solution was then aspirated, and 5 ml of Atto 488 NHS ester dye solution (Sigma-Aldrich, 41698) was added to the collagen gel and incubated at RT for 1 h. The dye solution was again aspirated and quenched in 10 ml of TRIS [tris(hydroxymethyl)aminomethane] buffer (Alfa Aesar, J62662) (pH 7.0) for 10 min at RT. Fluorescently labeled collagen gels were further washed six times in PBS (with Ca^2+^/Mg2^+^) over a 4-h period. The collagen gels were then depolymerized by adding 750 µl of 500 mM glacial acetic acid (Sigma, 45726) and placed on a rocker for 1 h at 4°C. Acidified collagen solution was then dialyzed (Thermo-Scientific, 87735) against 20 mM glacial acetic acid for 20 h at 4°C at a 1:1,000 ratio. The final labeled collagen concentration created was estimated by calculating the known starting and final volumes of the collagen solution (between 9 and 10 mg/ml). A total of 2–10% labeled collagen stock solution was then created by mixing with unlabeled type I collagen solution (calculations based on protein weight (Doyle, 2018)).

### Aligned 3D Collagen Hydrogel Preparation

To establish magnetic collagen type I hydrogels, anti-collagen I-labeled MNPs r non-labeled MNPs (Micromod, 09-02-0252), the desired concentration of MNPs was separated from aqueous suspension in a magnetic field generated by a permanent magnet. MNPs were then resuspended in ×1 DPBS (Gibco, 14190144) to obtain a final solution of 20% MNPs in DPBS. The pH of the collagen/MNP suspension was adjusted to pH 7.4 by adding 1 M NaOH (approximately 5% NaOH final concentration). The MNP suspension was then homogenized with a volume of fluorescent collagen solution (fluorescently labeled rat-tail collagen I and non-fluorescent rat-tail collagen I solution [Corning, 354249] at a ratio of 1:10) to get a final collagen I concentration of 3 mg/ml, and 20% HEPES-full DMEM was added to get the final 100 µl gel volume. Further to this, hASC (PT-5006, Batch 18TL212639) was resuspended in the collagen/MNP solution at a density of 2.5 × 10^4^ cells/ml prior to being aliquoted into each well of an eight-well chamber slide (Corning, 354118). The chamber was then placed between two N42 40 × 20-mm neodymium magnets (Bunting Magnetics, EP352), in parallel length-to-length 7.2 mm apart, resulting in a magnetic field strength of 50–52 mT. The north and south poles of each magnet were facing each other, and a magnetic field was established. Hydrogel-filled chamber wells were placed with the two magnets at an equal distance from the length of each magnet of the device. The chamber well slides were parallel to the length of the magnets. Simultaneously, the application of magnetic fibrillogenesis was initiated by incubating at 37°C for 30-min. Afterward, the magnetic field was removed, and gels were immersed in complete media (*α*-MEM, 10% fetal bovine serum, 1% penicillin–streptomycin) and further incubated at 37°C (5% CO_2_), elevated from a metal shelf present inside an incubator.

### Immunohistochemistry of 3D Collagen Hydrogels

A total of 3 mg/ml three-dimensional (3D) fluorescent collagen gels were prepared as previously described, with a cell density of 2.5 × 10^4^ cells. After culturing in complete media for 7 days (37°C, 5% CO_2_), the gels were fixed in 10% neutral buffered formalin (Sigma-Aldrich, HT501128) for 20 min, washed three times in PBS, and incubated in 0.1 M glycine in PBS for 5 min. Cells were then permeabilized in 0.1% Triton X-100 (Sigma-Aldrich, X100) in PBS for 3-min. Cellular actin filaments were immersed in rhodamine phalloidin (R415, Thermo Fisher) (1:2 phalloidin:PBS) for 50 min at RT. The gels were washed three times with PBS and then immersed in DAPI (4′,6-diamidino-2-phenylindole) (Thermo Scientific, 62248, 1:1000 DAPI: PBS) for 10 min at RT. The samples were washed three times with PBS, immersed in PBS, and imaged by confocal microscopy. If required, the gels were stored at 4°C in PBS.

### Image Analysis

To quantify the orientation of the fiber-like collagen structures, light and confocal microscopy images were analyzed with the Directionality plug-in for FIJI (Rezakhaniha et al., 2012; Schindelin et al., 2012). Fourier spectrum analysis provides a Gaussian distribution of the orientation of fiber-like structures (Liu, 1991; Schindelin et al., 2012; Schneider et al., 2012). The orientation is relative to the deviation from a transect across the width of the image, considered 0°. The measurement of the amount of structures was taken every 2° from 0 to 180°. Structures were considered anisotropic if a single isolated peak at a specific orientation could be identified. Orientation values were plotted as an average of the amount measured for three different gels, for each experimental group. The same analysis was adopted for the determination of hASC f-actin orientation, following the conversion of confocal images into a binary format.

The segmentation of the collagen I microarchitecture was also quantified through image analysis in FIJI (v2.3.0). Fiber-like structures are dependent on the presence of interspersing spaces within collagen. Therefore, the more frequent these gaps, the more fibrillar-like the structure. Plot profile analysis function of FIJI was used to provide a quantitative output of the 8-bit image. Transverse sections across the width of the image at half the measured height were used for analysis. A segment was defined as a width of the image in which the gray value was less than 25% the highest determined value and 15 μm or greater in length, determined by FIJI software. Three confocal images for each experimental group, taken at random, were averaged. For one sample in the non-magnetic gels, five measurements of gray value were disregarded due to the presence of an artifact (Supplementary Figure S1).

### 3D Magnetic Field and MNP Alignment Simulation

Finite element simulations (Comsol v5.1) were performed to demonstrate the behavior of MNP motion and their resulting orientation under the applied magnetic fields in which collagen hydrogels were incubated. The magnetic fields were simulated from two large N42 grade NdFeB permanent magnets positioned in the same orientations and distance apart as experimentally investigated. The dynamic behavior of MNPs was then simulated, using the particle tracing toolbox, through the interaction with the applied magnetic field through magnetophoresis (Eq. 1). MNP simulation parameters were matching those of the experimental use (250 nm diameter, density 5240 kg/m^3^, and susceptibility χ = 6.27). The fluid medium surrounding the MNPs was set to standard values of water to give an indication of particle motion.

$$F_{M}=2\pi r_{p}^{3}\mu_{0}\mu_{f}\frac{\mu_{p}-\mu_{f}}{{\text{μ}}_{p}+2\mu_{f}}\nabla H^{2},$$ (1)

where $r_{p}$ is the radius of the particle (in m), $\mu_{p}$ and $\;\mu_{f}$ are the permeability of the MNP and fluid, respectively (where μ=1+χ ), and ∇H is the magnetic field gradient (in Am^−2^) (Shiriny and Bayareh, 2020).

MNPs were initialized into the center of a cubic well in a fluid droplet with volume 100 µl and allowed to become randomly dispersed within the initial droplet. Following initialization, the particles were allowed to interact with the applied magnetic field with a time step of 0.01 s, and motion was allowed within the confined square culture well of size equaling that used experimentally. The resulting motion (shown in Supplementary Video S1) was analyzed by calculating the overall direction of motion (between end location and starting location). The angle of motion is described from the orthogonal to the applied field, similar to the measurement approach used for micrograph analysis.

### Immunofluorescent Staining of Tendomodulin and Scleraxis Proteins

Cellular 3 mg/ml collagen I gels were engineered as previously described (Methods 2.3). Following 30-min incubation at 37°C within a magnetic field, gels were submerged into complete media (*α*-MEM, 10% fetal bovine serum, and 1% penicillin–streptomycin). These cellular constructs were incubated at 37°C for 7 days, and media were replaced every 2 days as required.

Following incubation, gels were fixed as previously described (Methods 2.4). Gels were permeabilized and blocked simultaneously in 2% BSA/0.1% Triton X-100 (Sigma, X100) for 2 h at room temperature. Following incubation, permeabilization/blocking suspension was replaced with primary antibody suspension (anti-scleraxis [Abcam, ab58655] or anti-tenomodulin [Abcam, ab203676]) diluted at a 1:200 ratio in 1% BSA/0.1% Triton X-100. Control gels were immersed in 2% BSA/0.1% Triton X-100 without the addition of primary antibody. Gels were incubated overnight at 4°C with intermittent agitation. Suspension was removed, and gels were washed three times in wash buffer (x1 DPBS/0.1% Tween 20 [VWR, 663684B]). The gels were then immersed in secondary antibody (AlexaFluor 647, Invitrogen, A11037) diluted at a 1:1,000 ratio in 1% BSA/0.1% Triton X-100. The gels were incubated for 2 h at room temperature on a plate rocker in the dark. After that, the gels were washed three times with wash buffer and then counterstained by immersion in 0.02 μg/ml DAPI (Sigma, DP542) in deionized water for 20 min at room temperature with continuous rocking and protected from light. Counterstaining suspension was then removed, and the gels were washed three times in wash buffer. Cellular collagen I gels were visualized by confocal microscopy following immunofluorescent staining. Three images were taken per construct.

### Statistical Analysis

Analysis of the dose dependence of fiber-like structure growth was performed by a two-way ANOVA, adjusting for gel repeat as a covariate. Statistical analysis was performed in Stata v17 StataCorp (Texas, USA).

## Results

**FIGURE 1:**
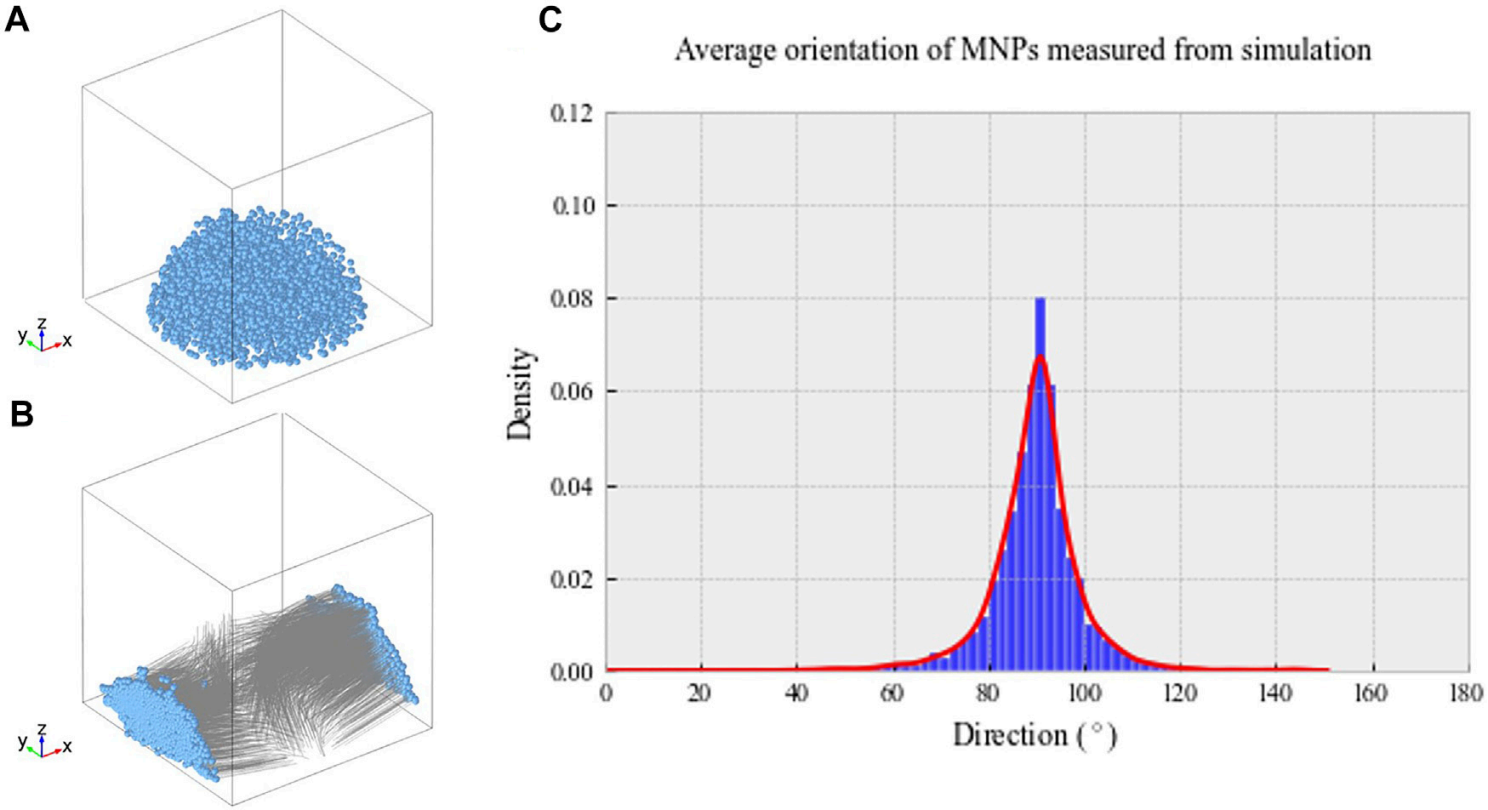
**(A)** Randomized MNP position within a model culture well plate at *t* = 0 s. **(B)** Final MNP positions at *t* = 0.1 s, with the magnetic field applied in the X direction. The MNP positions are indicated in blue, with gray lines demonstrating motion over the simulated time frame. **(C)** Average density of MNPs between 0 and 180° within our magnetic device simulated through computational modeling. Greatest MNP density was recorded at 90.33° aligned with the magnetic field, as indicated by the highest peak on the histogram.

Our data demonstrate the ability of anti-collagen I functionalized MNPs to guide collagen I fiber-like structure alignment *in situ* when a magnetic field is applied. Without MNP dosing or application of magnetic field, collagen gels did not align in the magnetic field. In addition, it was observed that uniaxially aligned, fibrillar hydrogel scaffolds provided topographical cues for the parallel alignment of hASCs *ex vivo.* Computational modeling predicted the MNP behavior within our hydrogels in the magnetic field. Simulations showed that hydrogel-laden MNPs align orthogonal to the applied field. The median density of MNPs was predicted to be at 90.33° from the applied field (Figure 1C). These tendon-mimetic constructs were immunofluorescently stained to visualize the expression of tendon-like cell markers.

### Simulated MNP Directionality

Computational modeling was implemented to predict hydrogel-laden MNP behavior within our magnetic array system (Methods 2.8). The magnetic field within the sample area was found to be 52.3 mT, similar to that found experimentally, and the field gradient acting upon the particles across the sample area was 25 T/m. Figure 1A shows the randomized initialization positions of MNPs, and Figure 1B shows the final MNP positions at *t* = 0.1 s. Within these simulations, the viscosity of the gels was not included; however, this would only affect the MNP velocity, rather than directionality. The median directionality was determined to be at 90.33° from the applied field (Figure 1C).

### MNPs Laden in Collagen I Hydrogels Can Be Manipulated Using an External Magnetic Field

**FIGURE 2:**
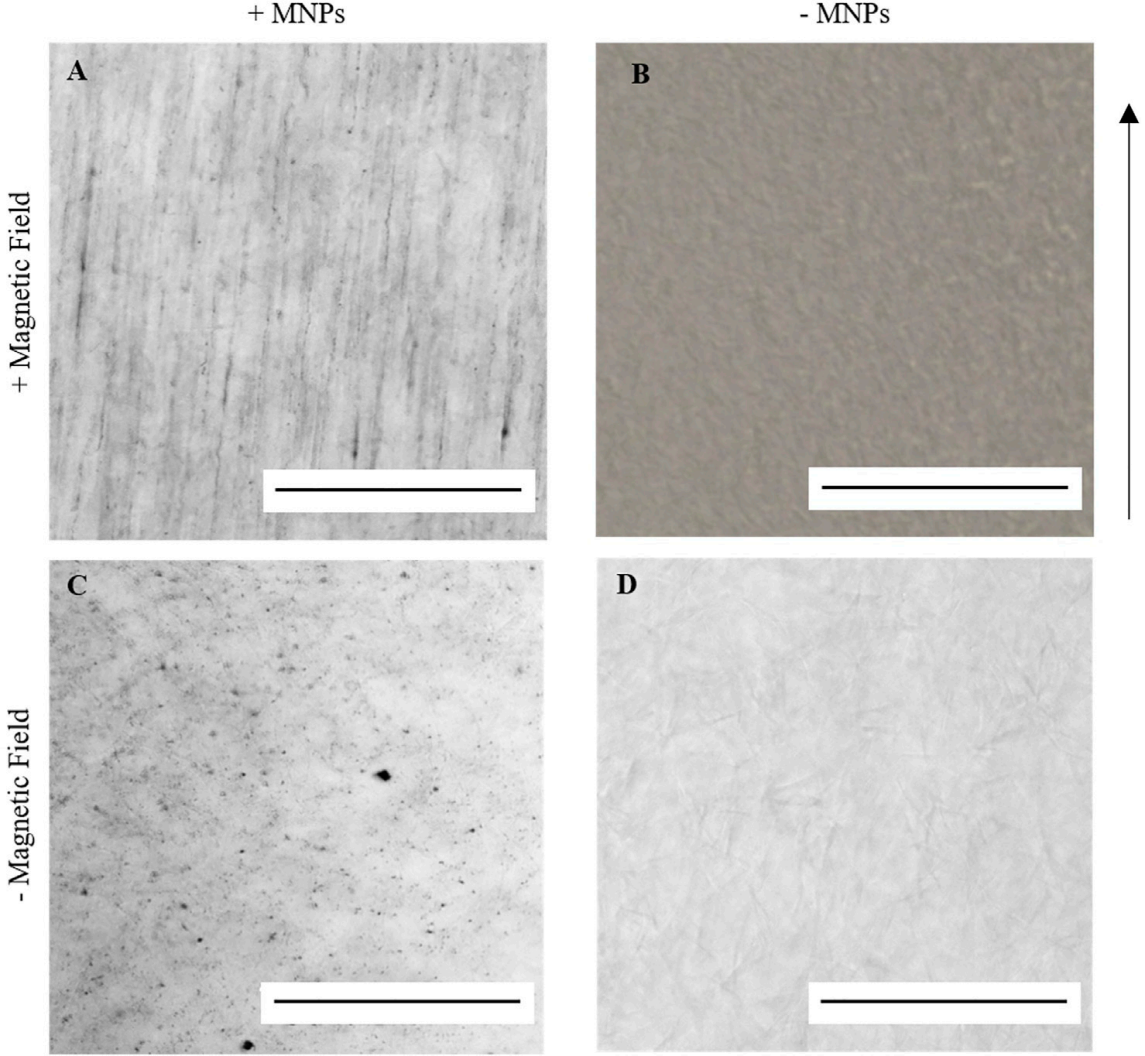
Light microscopy imaging of 0.5 μg/μl 250 nm dextran-coated MNPs in 100 μl 3 mg/ml collagen I gels. A and C = MNP-laden gels; B and D = gels without MNPs. When gelled in the presence of an external magnetic field at 37°C for 30 min, string-like structures could be identified in MNP-laden gels **(A)** but not in non-magnetic gels **(B)**. When gelled at 37°C without the application of the magnetic field, these structures could again not be identified **(C,D)**. Scale bar = 0.5 mm, magnification = ×20, arrow = direction of magnetic field, and *n* = 3.

When encapsulated within an aqueous collagen solution (3 mg/ml), 250-nm dextran-coated MNPs (0.5 μg/μl) were evenly dispersed throughout the collagen suspension. Following 30-min incubation within a magnetic field, MNPs align and form string-like structures visible under light microscope in the presence of a magnetic field (Figure 2A). Aligned MNPs were not observed when the magnetic field is not present (Figure 2C). Collagen gels without MNPs (hereafter “non-magnetic gels”) do not demonstrate orientated MNP alignment with (Figure 2B) or without (Figure 2D) the magnetic field. Our study demonstrates the ability to remotely control MNP orientation in collagen I hydrogels using an external magnetic field for 30 min at 37°C.

To note, the polymerization of collagen atop a metal shelf within a 37°C incubator resulted in decreased collagen alignment against the magnetic field. It has been previously noted (Guido and Tranquillo, 1993; Shannon et al., 2015) that by applying a constant magnetic field, the total energy needed for MNP-facilitated uniaxial collagen orientation must be greater than the thermal energy applied for collagen fibrillogenesis to occur. Therefore, polymerized collagen was maintained on polystyrene to prevent contact with the incubator metal shelving, in turn decreasing the rate of fibrillogenesis and enhancing the magnetic alignment of the collagen fibers (Guido and Tranquillo, 1993; Shannon et al., 2015).

### Anti-Collagen I Functionalization of MNPs Facilitates MNP-Directed Collagen Alignment

**FIGURE 3:**
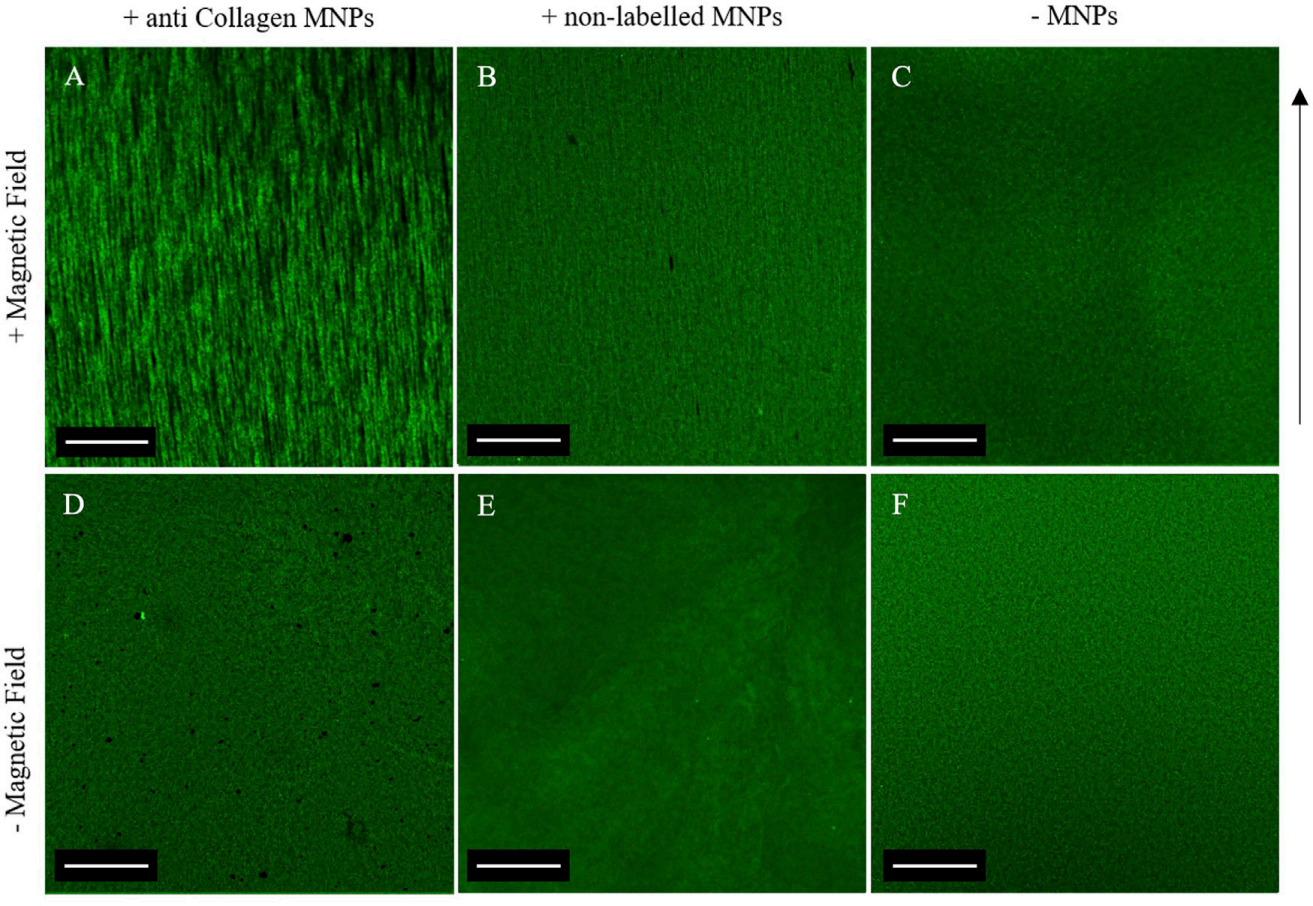
Confocal microscopy of 0.5 μg/μl anti-collagen MNP-laden fluorescent gels **(A,D)**, 0.5 μg/μl non-labeled MNP-laden fluorescent gels **(B,E)**, and non-magnetic fluorescent gels **(C,F)**. Top panel: gels post-application of the magnetic field. Bottom panel: gels with no magnetic field application. A clear striated macroscale organization is only evident when a magnetic field applied to anti-collagen MNPs **(A)**. In all other gels, a disorganized matrix is shown, and the anisotropic topography is absent. Scale bar = 1 mm, magnification = ×4, green = collagen I, arrow = direction of magnetic field lines, and *n* = 3.

Following confirmation that collagen-encapsulated MNPs can be manipulated *in situ* within a magnetic field, the effect of the aligned MNPs on the collagen hydrogel microstructure was determined. We hypothesized that MNPs with an applied magnetic field modulate topography of collagen hydrogels as they migrate, causing segmentation of the gel and resulting in fiber-like structures that align in parallel to MNP orientation. Fluorescently tagged collagen fibrils could be visualized under a confocal microscope (Figure 3). Despite the orientation of MNPs following magnetic field lines (Figures 1, 2A), parallel striation of collagen topography was less pronounced (Figure 3B). To overcome this, we functionalized 250-nm dextran-coated MNPs with anti-collagen I antibody (hereafter “anti-collagen MNPs”) to determine if this would enhance MNP-facilitated collagen alignment. A total of 3 mg/ml collagen I hydrogels were laden with 0.5 μg/μL anti-collagen MNPs (hereafter “anti-collagen MNP gels”) and evident striation of collagen was observed, resulting in fiber-like structures (Figure 3A). This segmentation was evident throughout the entire 3D structure (Supplementary Video S2). In non-magnetic gels subjected to a magnetic field (Figure 3C) and constructs gelled without the presence of an external magnetic field (Figures 3D–F), only randomly orientated fiber-like collagen structures could be observed, with no striated topography. It can therefore be concluded that collagen topography can be manipulated by the magnetic field, dependent upon MNPs.

**FIGURE 4:**
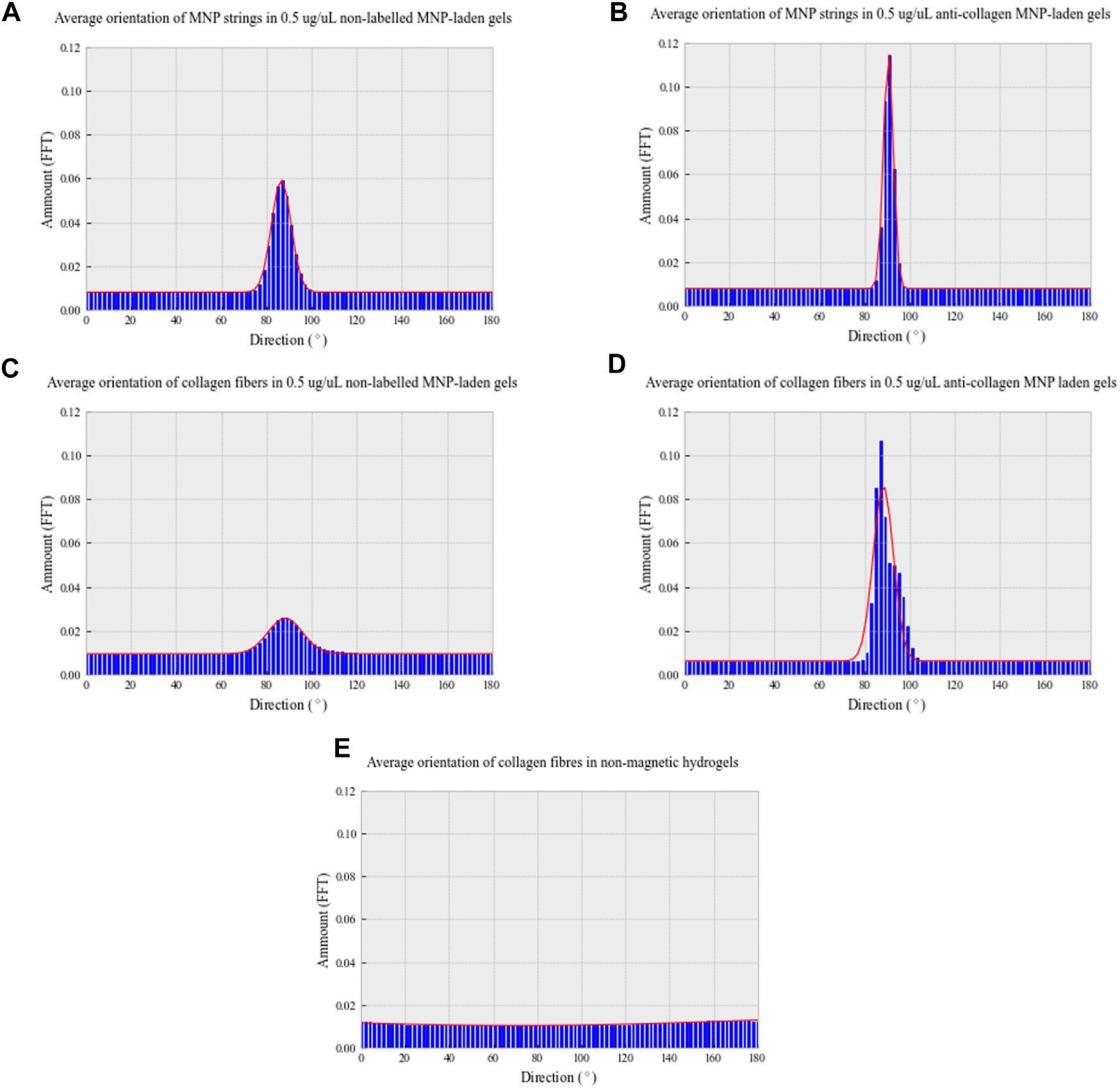
Directionality distribution histograms of 0.5 μg/μl non-labeled MNP strings **(A)** and fiber-like collagen structures **(C)**; 0.5 μg/μl anti-collagen MNP strings **(B)** and fiber-like collagen structures **(D)**; non-magnetic **(E)** collagen gels. All gels were exposed to a 30-min magnetic field **(E)**. To determine MNP and fiber-like collagen structure alignment, light microscopy and confocal microscopy images, respectively, were analyzed with Directionality plug-in of ImageJ in Fourier spectrum analysis mode. *n* = 3, trendline = fit to the Gaussian function.

Analysis of both light and confocal microscopy data was undertaken to compare the orientation of MNPs and directionality of collagen hydrogels. Fiber-like collagen structures would be anticipated to result in anisotropic topography, comparable to MNP orientation. Non-labeled and anti-collagen MNPs were most frequently orientated between 86.6° and 90.5°, respectively (Figures 4A,B). This behavior is consistent with computational simulations of collagen-laden MNP motion within our magnetic device system, which is orientated at 90.33° (Figure 1). The peak frequency of collagen orientation was determined to align at 88.3° in both non-labeled and anticollagen MNP gels. However, a larger variability was noted within the non-labeled MNP gels (SD ± 3.232) (Figure 4C) compared to anti-collagen MNP gels (SD ± 1.9) (Figure 4D). Considering this variation, anti-collagen gels orientate at a degree most closely to MNPs, from both modeling and experimental data. Last, no single directionality within fiber-like collagen structure orientation was determined within the non-magnetic collagen gels (Figure 4E).

From these data, we conclude that using our magnetic device, we are able to induce the alignment of MNPs that has preferred directionality relative to MNP orientation. Therefore, anti-collagen MNP gels were selected for use in further experiments. Without MNP dosing or the application of magnetic field, collagen is randomly orientated, resulting in isotropic structures with no preferred directionality. Next, we hypothesized that increasing MNP dosage will alter the segmentation of fiber-like collagen structures.

### Magnetic-Dependent Orientation of Collagen I Fiber-Like Structures Is Dose Dependent With MNPs

**FIGURE 5:**
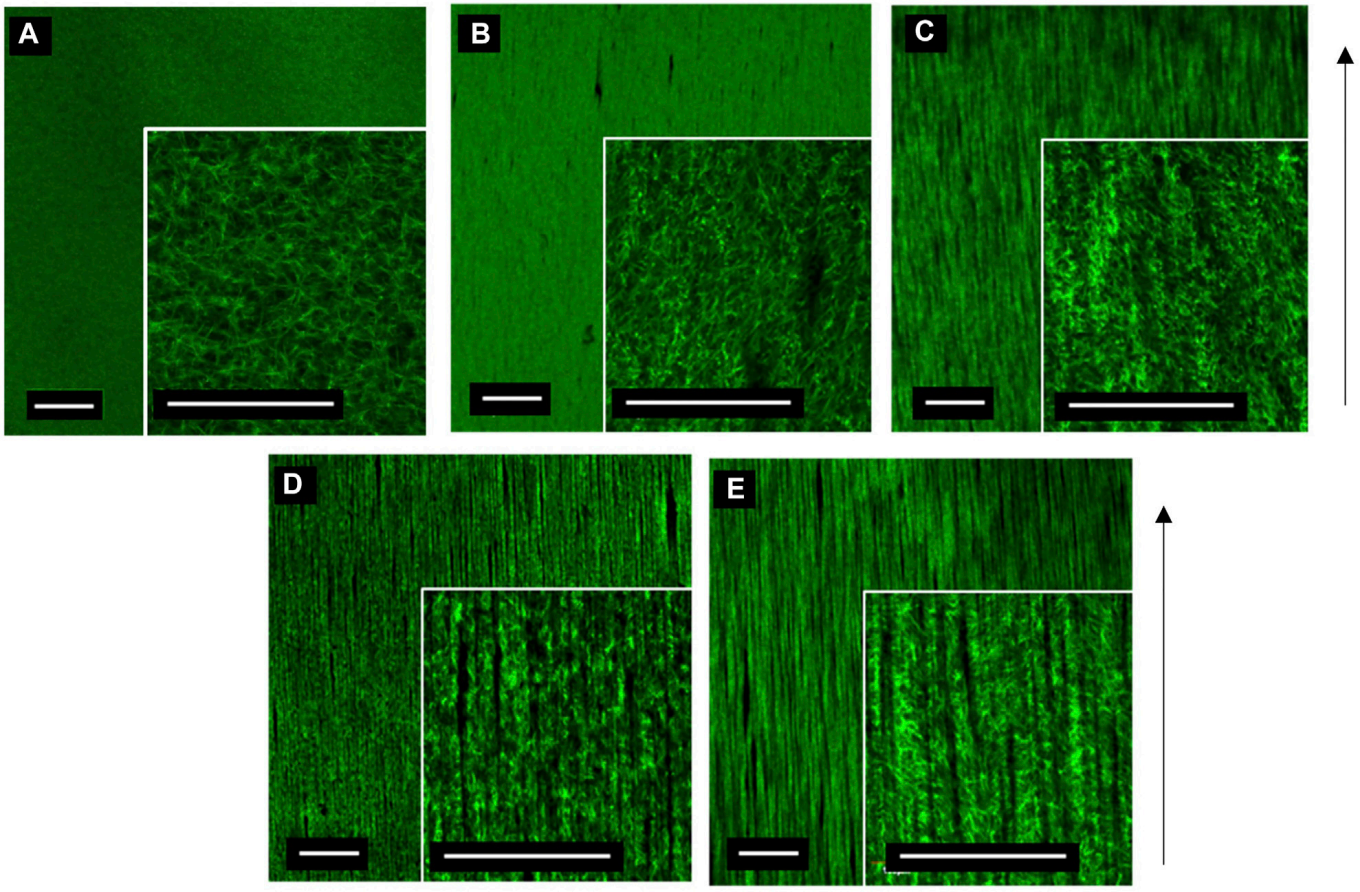
Confocal microscopy images of 3 mg/ml collagen hydrogels dosed with control no MNPs **(A)**, 0.25 **(B)**, 0.5 **(C)**, 0.75 **(D)**, and 1 μg/μl **(E)** anti-collagen MNPs held within a magnetic field for 30 min during collagen gelation (taken at day 0). Scale bar = 0.5 mm, magnification = ×4 and 20× (inner panels), green = collagen I, arrow = direction of magnetic field lines, and *n* = 3.

The relationship between MNP dosing and segmentation of collagen hydrogels was determined. A total of 3 mg/ml collagen gels were prepared with increasing MNP concentrations (0.25–1.0 μg/ml) of anti-collagen MNPs and visualized post-incubation in the presence of a magnetic field (Figure 5). As anti-collagen MNP dosing increases, so does the occurrence of collagen segmentation, resulting in anisotropic fiber-like structures visible in 0.50, 0.75, and 1.00 μg/μl anti-collagen MNP-dosed gels (Figures 5B–D). Images were analyzed quantitatively to compare the segmentation of collagen hydrogels in response to MNP dosing.

**FIGURE 6:**
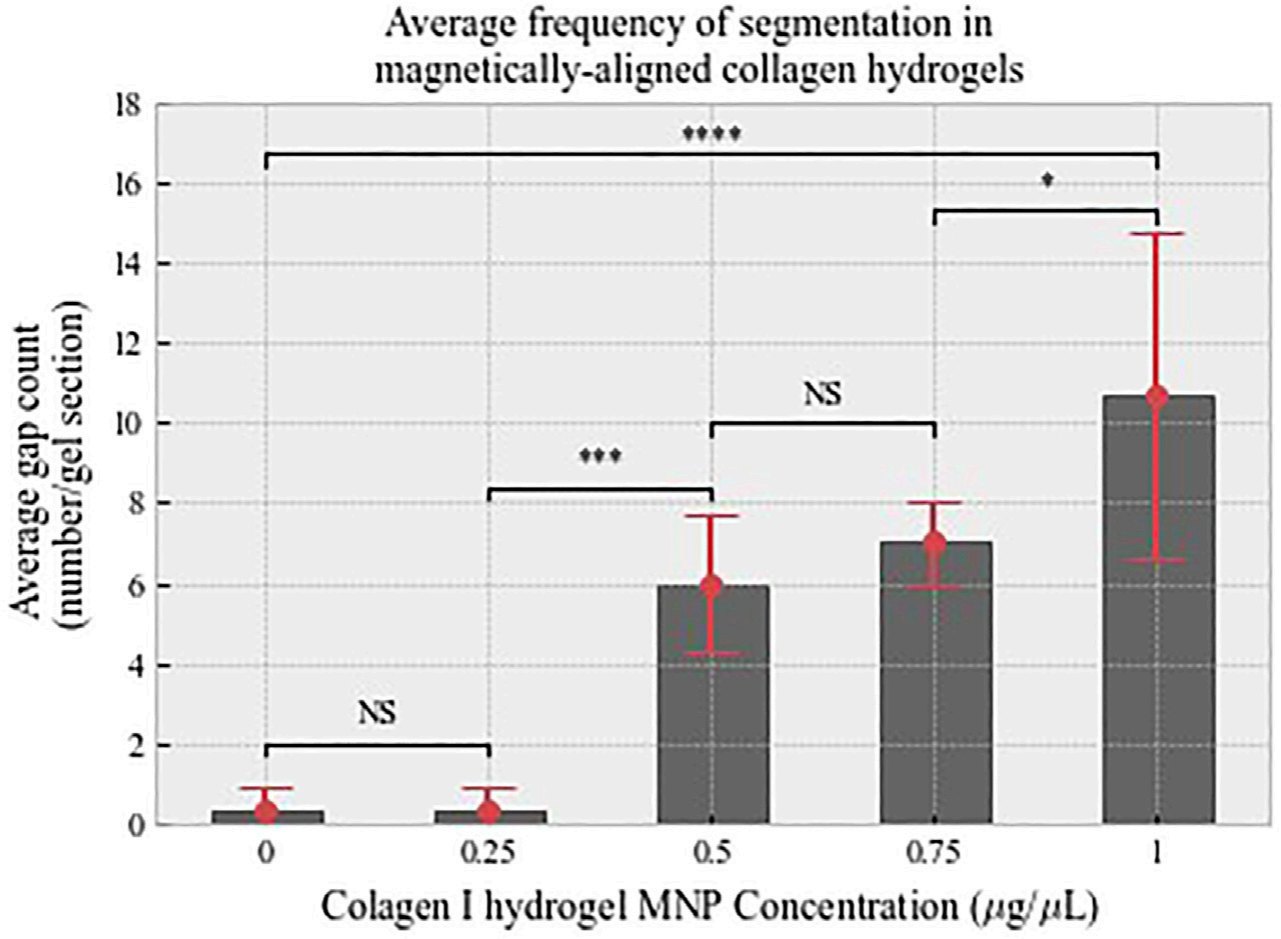
Average count of interspersing gaps that define fiber-like structures analyzed from confocal data. There is a positive relationship between MNP dosing and segmentation. *n* = 3, error bars show standard deviation. Shown levels of significance *p* < 0.001 is denoted by ***, and *p* < 0.0001.

Quantitatively, collagen segmentation is represented by areas in which there is a decrease in the gray value output from plot profiling of confocal images (Methods 2.5). There is a positive correlation between MNP dosing and frequency of segmentation (Figure 6). Then 0.00 μg/μl (non-magnetic) and 0.25 μg/μl MNP-dosed collagen gels were not found to be segmented, and thus, fiber-like structures were absent (Figure 6). There is a significant difference between segmentation frequency in 1.00 μg/μl anti-collagen MNP-dosed gels compared to 0 μg/μl (*p* < 0.0001), with smaller significance levels found between differing concentration values (Figure 6). This organization was observed to persist for at least 21 days (Supplementary Figure S2), and average 1.00 μg/μl MNP-dosed hASC viability was determined to be 97.83% when normalized to control groups (Supplementary Figure S3). In total, 0 μg/μl and 1 μg/μl dosed gels were selected for further experiments in which we establish cellular isotropic and anisotropic collagen I hydrogels, respectively, to determine the effect of topography on cell alignment. No relationship between MNP concentration and width between fiber-like structures could be determined.

### MNP-Facilitated Anisotropic Collagen Hydrogels Provide Topographical Cues for hASC Culture

**FIGURE 7:**
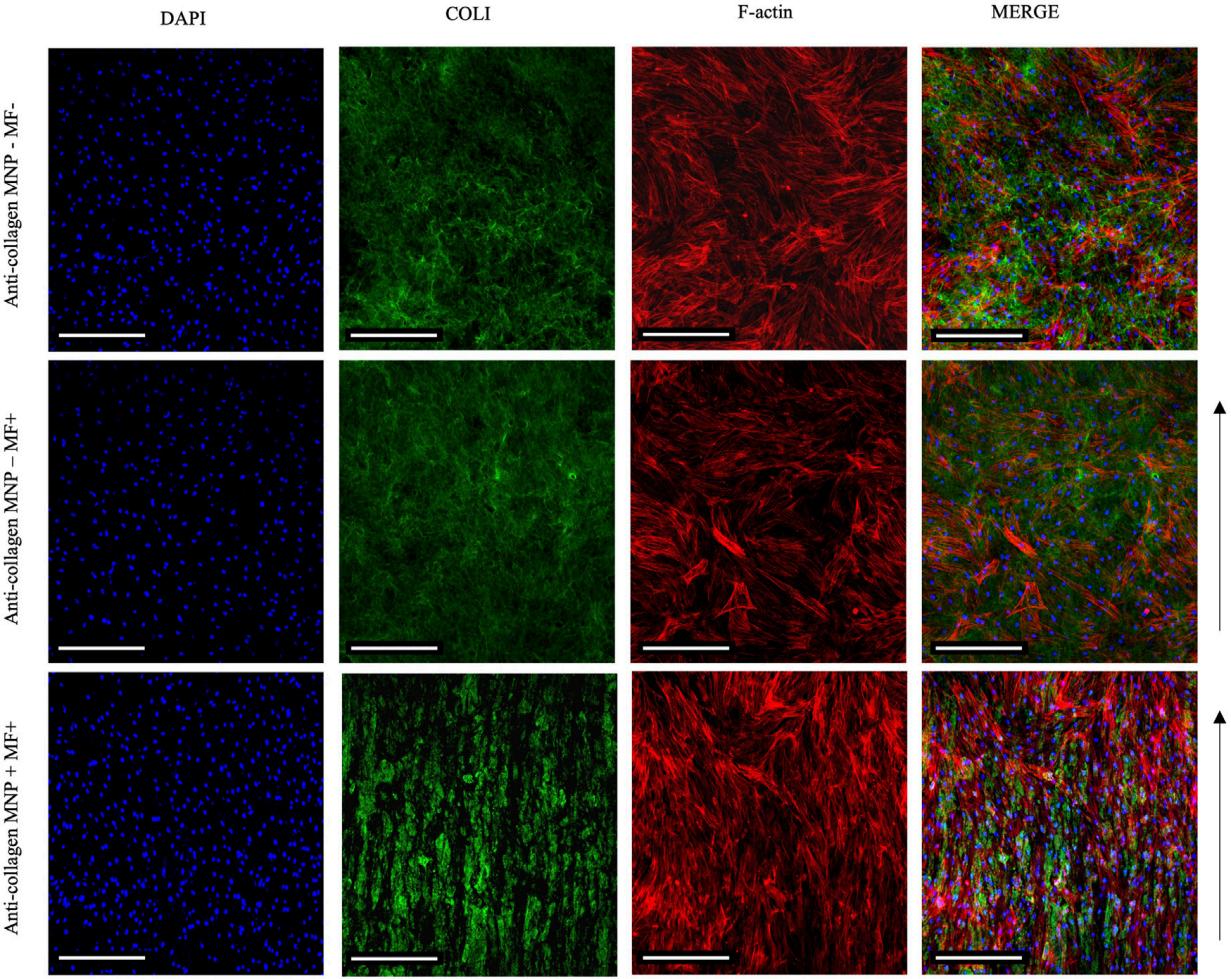
Confocal imaging of primary hASCs seeded (250 cells/ul) within collagen hydrogels 7 days post-seeding. Merged images show an aligned arrangement of hASCs, in parallel to collagen fiber-like structures in anti-collagen MNP-laden, magnetically aligned gels (bottom panel). Without MNP dosing and/or application of the magnetic field, f-actin orientation can be observed to be poly-directional (top and middle panels). Scale bar = 200 μm, magnification = ×10, blue = nuclei, red = F-actin, green = collagen I, arrow = direction of magnetic field lines, and *n* = 3.

In our optimized protocol, we utilized anti-collagen MNPs subjected to a magnetic field to facilitate the formation of anisotropic collagen matrices. The absence of MNP dosing resulted in isotropic structures to be observed. Exploiting this, we established isotropic and anisotropic cellular hydrogels seeded with hASCs to investigate the effect of the matrix architecture on cell organization. We hypothesized that 1) magnetically aligned collagen hydrogels would facilitate parallel, unidirectional cell organization, and 2) non-magnetic hydrogels—both with and without the application of magnetic field—would facilitate poly-directional cell organization. Prior to these experiments, we confirmed that an MNP dosing of 1 μg/μl had little effect on cellular viability (Supplementary Figure S3), suggesting that MNPs did not present any cytotoxic effects on the hASC, further confirming the appropriateness of the protocol for physiologically relevant models. Following 7 days in culture, magnetically aligned and non-aligned cellular hydrogels were fixed and immuno-stained so that cell nuclei and actin filaments could be observed through confocal microscopy (Figure 7).

**FIGURE 8:**
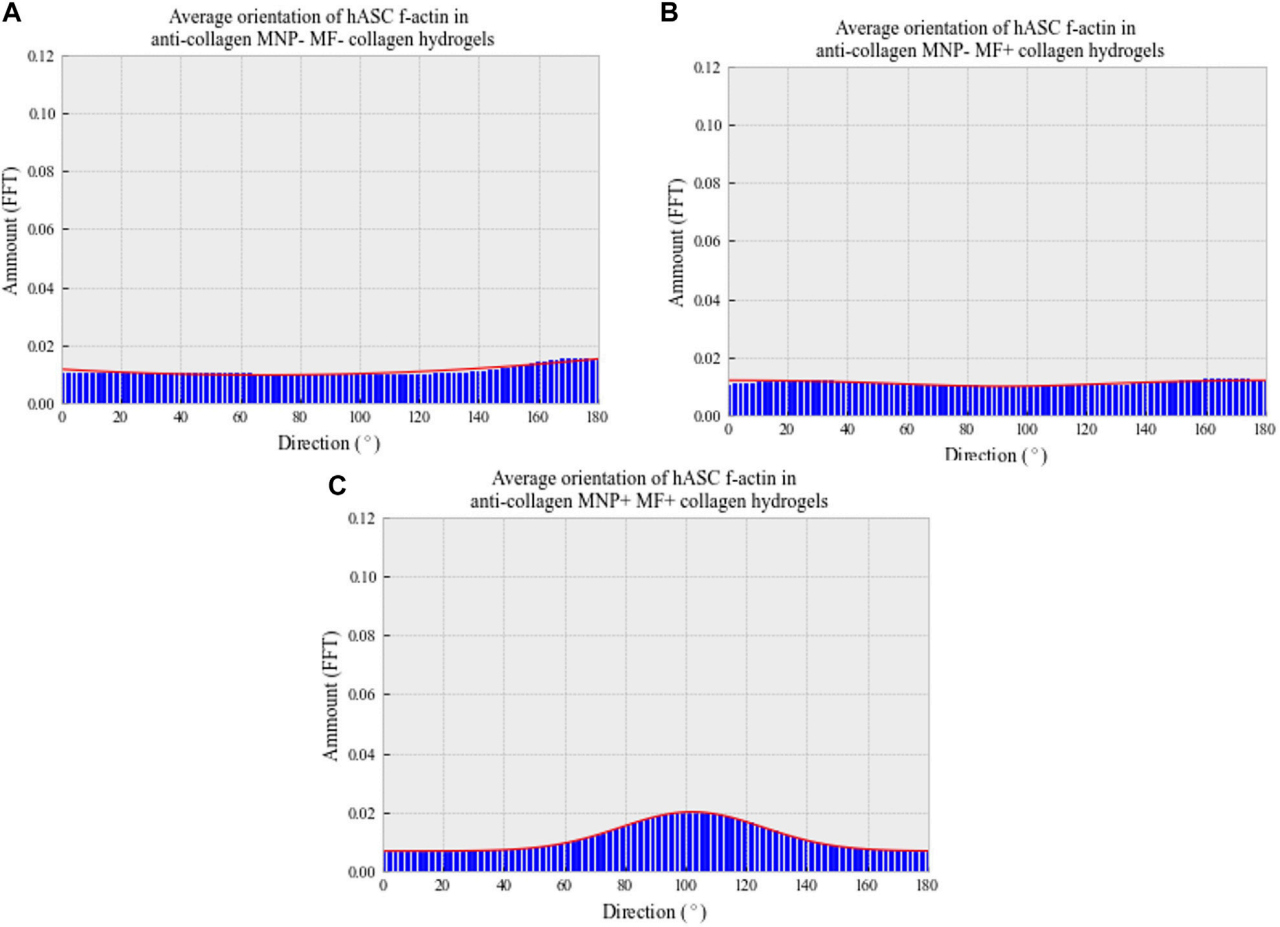
Average directionality histograms of hASC f-actin 7 days post-gelation in collagen hydrogels. Patient-isolated hASCs (passage 4) were seeded (250 cells/μl) into depolymerized collagen suspensions, either with **(C)** or without **(A,B)** anti-collagen MNP dosing. Cell–collagen suspensions were then gelled and, if appropriate, a magnetic field applied for 30 min at 37°C. Hydrogels were then immersed in basal media and fixed following 7-day incubation. F-actin was visualized using confocal microscopy, following rhodamine staining. Orientation determined through ImageJ’s Directionality plug-in, and averages plotted. *n* = 3, trendline = fit to the Gaussian function.

In MNP-dosed hydrogels subjected to a magnetic field, cellular F-actin filaments were observed to be unidirectional in their orientation, aligning parallel to the direction of the fiber-like collagen structures and MNPs (Figure 7 bottom panel). In non-magnetic collagen gels, cytoskeletal structures were orientated in multiple directions, regardless of the presence of magnetic field (Figure 7 top and middle panels). Further to this, we quantified cell orientation in relation to the applied magnetic field to compare the effect of MNP dosing on hASC organization (Methods 2.5). Plotting of the average amount of hASC aligning at 0–180° results in a single, normally distributed peak is shown in Figure 8C). This demonstrates that the cells are aligning at a preferred orientation. Such preference of hASC orientation cannot be determined when laden in non-magnetic collagen gels, regardless of the presence of the magnetic field (Figures 8A,B). This is consistent with the visual assessment of the hydrogels (Figure 7). From these data, we conclude that we are able to remotely control and direct hASC alignment through MNP-mediated modulation of the collagen hydrogel architecture after seeding.

### Collagen I Scaffolds Harnessed Tendon-like Cell Population

**FIGURE 9:**
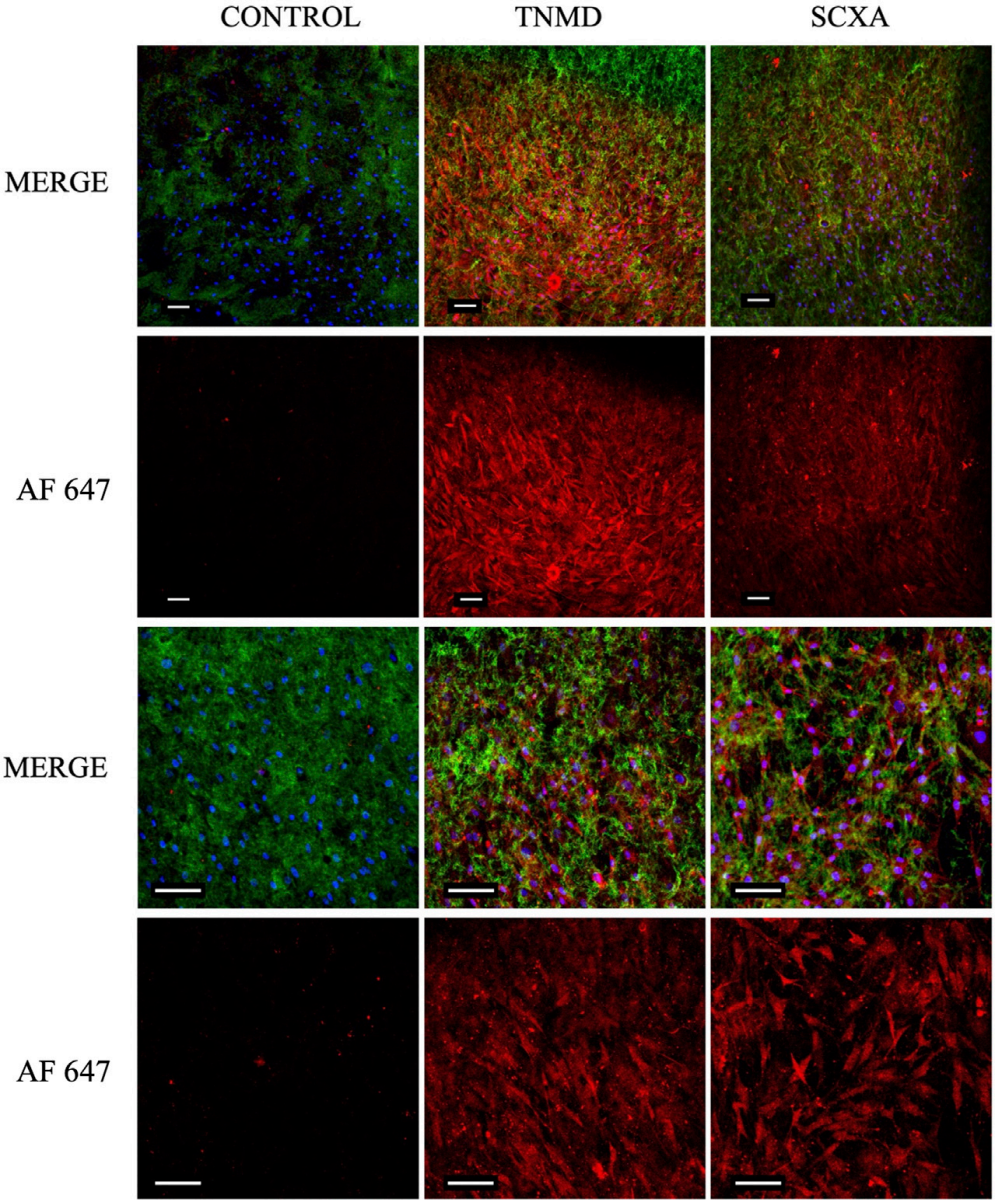
Confocal images of cellular collagen I scaffolds 7 days post-gelation following fixation and immunofluorescent staining for TNMD and SCXA. Expression of both proteins could be visualized in the experimental groups but not in the controls, indicating the tendon-like nature of the embedded cells. Scale bar = 100 μm, magnification = ×10 (rows 1 and 2), ×20 (rows 3 and 4). Blue = nuclei, green = collagen I, red = TNMD or SCXA (AF 647 = Alexa Fluor 647), and *n* = 3.

Once hASC–collagen fiber parallel alignment had been achieved, we looked to characterize the tenogenicity of the cell population. Following 7-day incubation in collagen I scaffolds, hASCs were immunofluorescently stained to identify the expression of tenocyte-associated proteins tenomodulin (TNMD) and scleraxis (SCXA). Expression of both TNMD and SCXA proteins was observed in experimental groups and absent from the control groups (Figure 9), with a significantly greater fluorescent intensity of the experimental groups compared to the controls (Supplementary Figure S4), which ensured the successful antibodies’ specific binding. Furthermore, staining patterns characteristic of cellular morphology comparable to the observations of hASC actin filaments were only present in experimental groups (Figures 7, 9). From this, we could conclude that tenocyte-like cells were harnessed in our collagen I scaffolds.

## Discussion

While approaches such as 3D printing have advanced the development of human-relevant 3D cell culture systems, challenges in mimicking the natural environment still remain. A particular barrier to engineering biomimetic *ex vivo* tissues is the fine-tuning and modulation of the ECM architecture, especially after cell-seeded constructs have been formed. The use of nanomaterials such as MNPs to achieve this, which have been used extensively in medicine, would be desirable (Abdulghani and Mitchell, 2019). In this work, we have presented a novel biocompatible, non-invasive approach that allows remotely controlling collagen type I hydrogel alignment *in situ* for tendon modeling and repair.

We have demonstrated the ability to remotely control MNP orientation in collagen type I hydrogels when exposed to a magnetic field (Figure 2). The alignment of both matrices and cells using magnetic approaches has been proposed in the neuronal field, in which oriented collagen gels were investigated for directed neuronal growth (Antman-Passig and Shefi, 2016), and magnetic fields have been used to direction neurite fiber outgrowth (Bongaerts et al., 2020). In this study, we have demonstrated the relevance of this approach to tendon tissue engineering where alignment of collagen fibers is a key element in ECM organization. We have further shown that collagen fibers can be orientated in a banded structure using this approach and that adult-derived stem cells (hASC) align along these banded regions. The alignment of MNPs and collagen type I in the magnetic field enables a unidirectional isotropic organization which establishes a tendon-like morphology. Ultimately, we would propose that this template would then drive downstream tendon growth and remodeling, which requires further study. The labeling of MNPs embedded in the gel with an anti-collagen I antibody was useful to enhance the directionality of both the MNP strings and the collagen fibers. MNP strings orientation changed from 86.6° to 90.5°, more in line with the computational modeling of the MNP alignment when exposed to a magnetic field, and the collagen fiber orientation distribution became more constant with a peak at 88.3°. Further study would examine the impact of the collagen hydrogel viscosity on MNP and collagen alignment.

Segmentation of the collagen hydrogel, as defined in Paragraph 3.4, is affected by MNP dosing; in particular, there is a positive relationship between the increment of MNP concentration and the frequency of interspacing gaps defining fiber-like structures. A total of 1 μg/μl anti-collagen MNP concentration was found to be optimal to establish fiber-like collagen structures. Exploiting this, we demonstrate how the MNP-mediated anisotropic collagen gel architecture facilitates directed, parallel hASC alignment. hASCs’ actin filaments orient along the collagen fibers and MNPs, into an organized complex structure that is not observed in collagen gels without MNPs and/or magnetic field exposure. Cellular characteristics, such as morphology and orientation, have been evidenced to be mediated by signaling pathways modulated in response to topographical cues (Andalib et al., 2013; Abdulghani and Mitchell, 2019). Therefore, controlling matrix topography would enable control of such characteristics in engineered tissues. Musculoskeletal tissues, including tendon, are reliant on the specific organization of ECM components, mediated and sensed by resident cells. Healthy native tendon is characterized by a hierarchical fibrillar cellular matrix, which provides elasticity and mechanical properties of the tissue, essential for its load transferring function (Screen, 2008). In tendinopathy, loss of this ECM organization and transition to isotropy is a key marker of this disease state (Bedi et al., 2012). Therefore, in order to engineer *ex vivo* constructs analogous to tendon tissue for disease modeling and regenerative therapies, tuning of ECM is paramount.

Our work in establishing a novel, non-invasive way of fine-tuning topographical characteristics of biomaterial-based 3D *in vitro* models represents a promising development in the field of tissue engineering and organ on a chip. We were able to engineer constructs comparable in gross organization to the hierarchical, fibrillary-like structure of healthy native tendon which could be generated after construction within an engineered tissue or an organ on a chip device. Emulating this *ex vivo* is key to unlocking the potential of regenerative therapeutics to address the outstanding clinical need posed by tendinopathies and other musculoskeletal disorders in which biomechanics are imperative to physiology.

Seven days post-gelation, immunofluorescent staining of embedded cells revealed expression of both TNMD and SCXA in collagen scaffolds. The transmembrane glycoprotein TNMD (Hou et al., 2017) has been found to be imperative to the development and maturation of tenocytes (Shukunami et al., 2016). Its expression is linked and activated by SCXA (Shukunami et al., 2018) which constitutes, together with TNMD, a marker of tenocyte-like cells (Komura et al., 2020). Within our tendon-mimetic system using adipose-derived stem cells, we observed the expression of both TNMD and SCXA after differentiation within our aligned gels.

It has previously been shown that stem cell therapies offer a desirable therapeutic option for tendon repair (Buzhor et al., 2014; Gaspar et al., 2015). The tenogenic potential of hASCs has been previously evidenced (Yee Lui, 2015). Furthermore, Hou et al. (2017) demonstrated the effectiveness of TNMD-expressing stem cells in facilitating tendon repair. It would be desirable to determine if tenocyte-associated protein expression observed in our tenogenic model is influenced by the orientation of collagen, and if orientation further modulates collagen synthesis or remodeling. This would be useful to optimize the model for its potential application in tendon repair.

To summarize, a biomimetic tendon microenvironment has been engineered for potential applications toward *in vitro* modeling of healthy and diseased tendons and informing the generation of tendon implants for the repair of pathological tissue.

## Data Availability

The raw data supporting the conclusions of this article will be made available by the authors, without undue reservation.
