# Supplementary figures and images for: Magnetic Nanoparticle-Mediated Orientation of Collagen Hydrogels for Engineering of Tendon-Mimetic Constructs
Source: Front Bioeng Biotechnol. 2022 Mar 17;10:797437. doi: 10.3389/fbioe.2022.797437 (PMC8968910; doi:10.3389/fbioe.2022.797437)

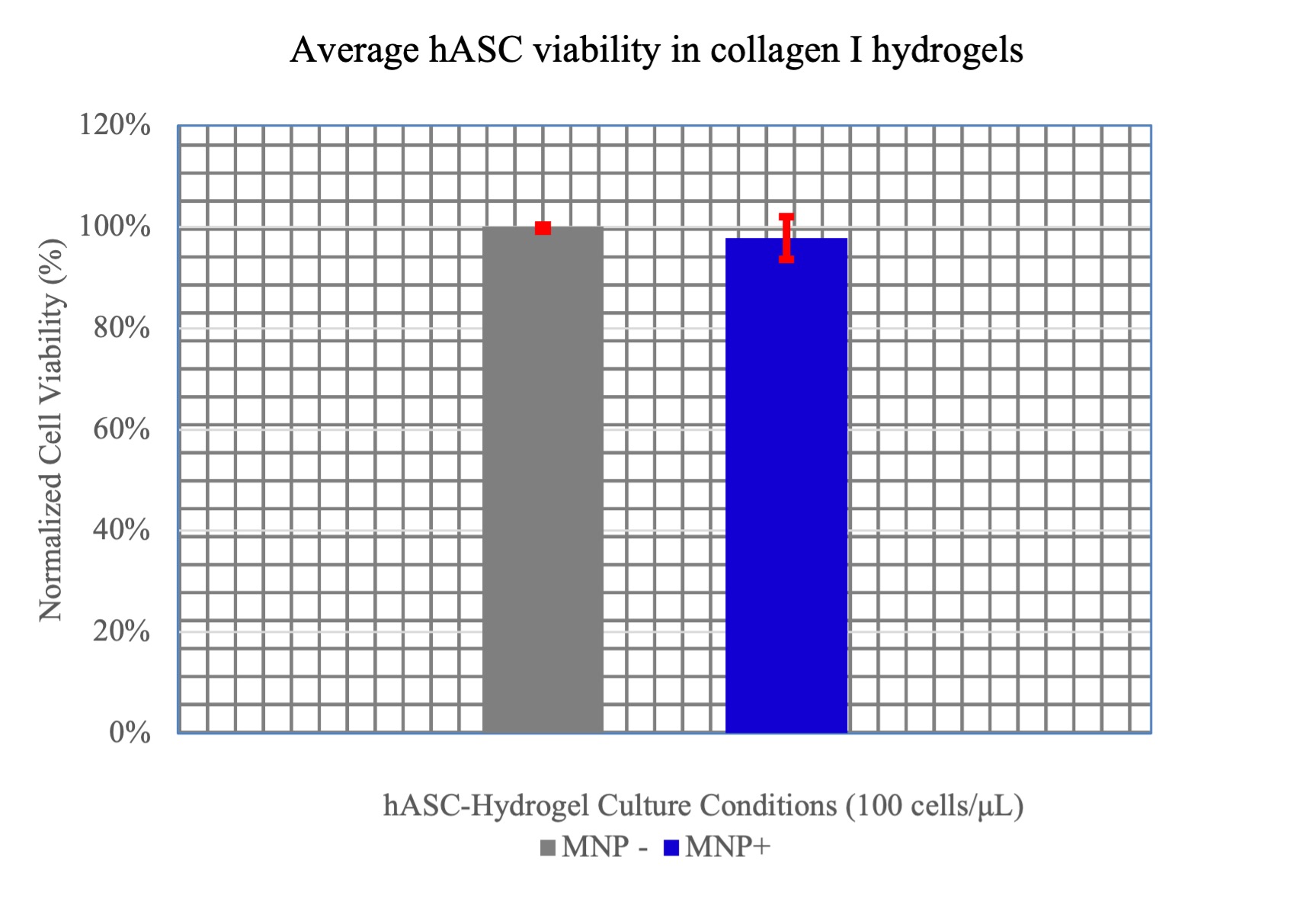

Supplement: Supplementary file 2 [file Image3.JPEG]

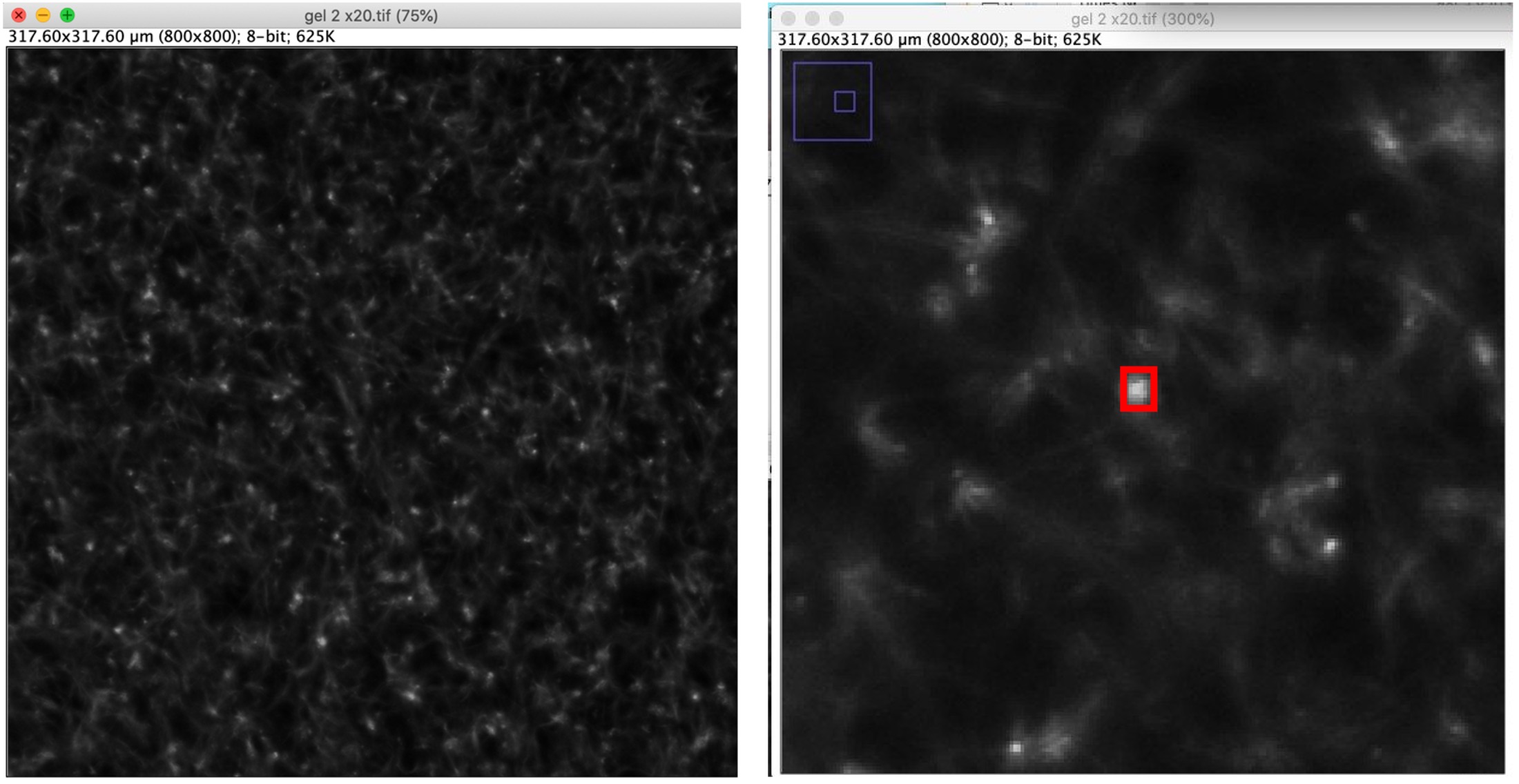

Supplement: Supplementary file 3 [file Image1.JPEG]

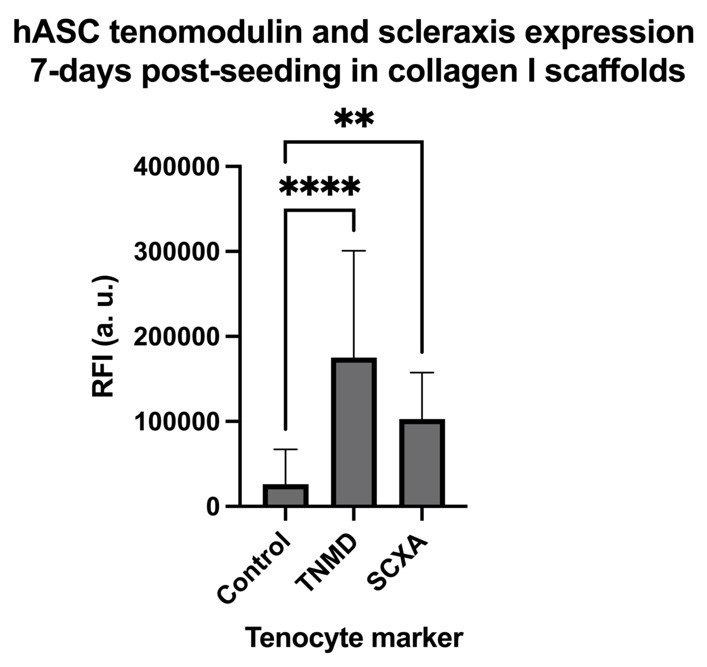

Supplement: Supplementary file 4 [file Image4.JPEG]

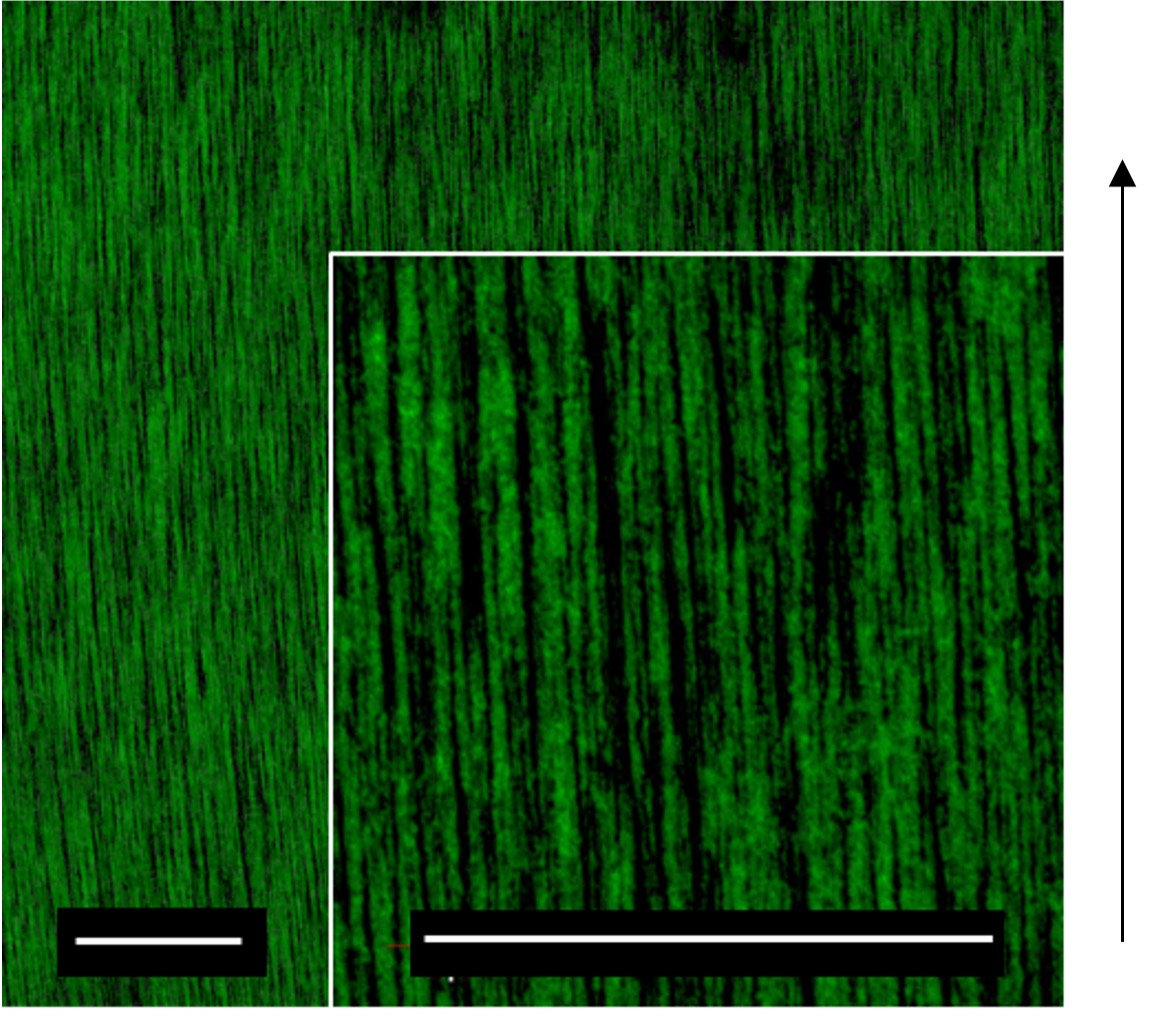

Supplement: Supplementary file 5 [file Image2.JPEG]
