# Supplementary material for: Magnetic Nanoparticle-Mediated Orientation of Collagen Hydrogels for Engineering of Tendon-Mimetic Constructs
Source: Front Bioeng Biotechnol. 2022 Mar 17;10:797437. doi: 10.3389/fbioe.2022.797437 (PMC8968910; doi:10.3389/fbioe.2022.797437)
Supplement: Supplementary file 7 [file DataSheet1.DOCX]

Supplementary Material

# Supplementary Data

#
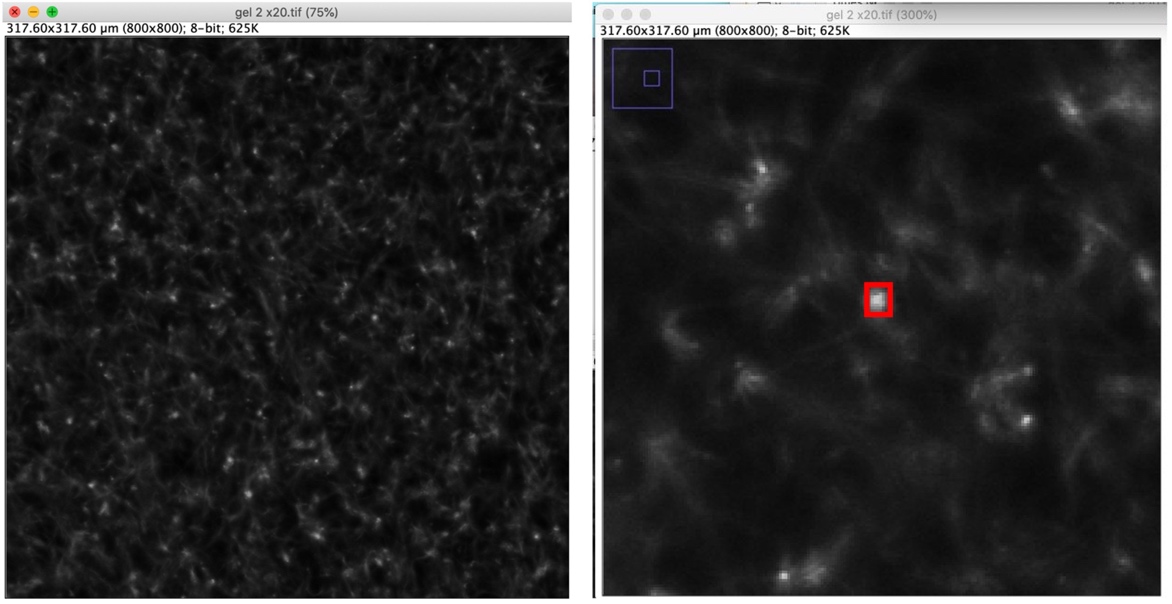


**Supplementary Figure 1.** Screenshot of non-magnetic gel with magnification of artefact present in the transverse section analyzed for segmentation analysis. This is the result of aggregation of fluorescent collagen that has not been homogenously dispersed in the suspension. This gives rise to a high grey value for this area, which is 5 microns in length. Therefore, grey value measurements taken at this point indicated in red were excluded from the determination of the highest grey value during image analysis.

**
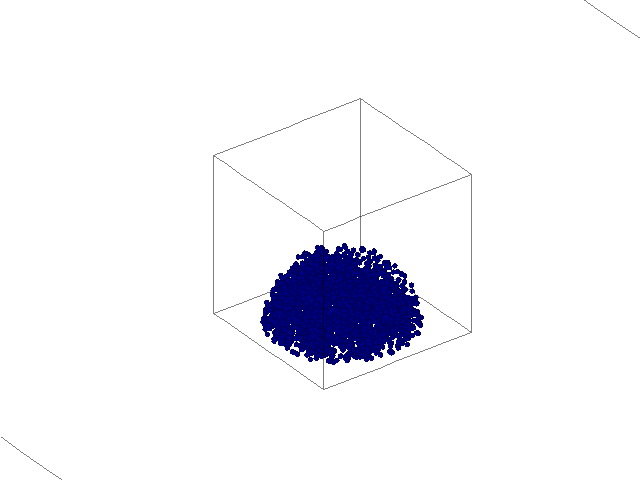
**

**Supplementary Video 1.** Simulation of Video of MNPs moving when magnetic field applied from our device. The majority of MNPs travel along the applied field, travelling to opposite sides of the sample area due to the field gradients. A small number of particles travel in a slightly curved trajectory if they were initialised close to the centre of the sample region. This is due to the y-component of the field gradient being non-zero and almost the same as the x-component of gradient.


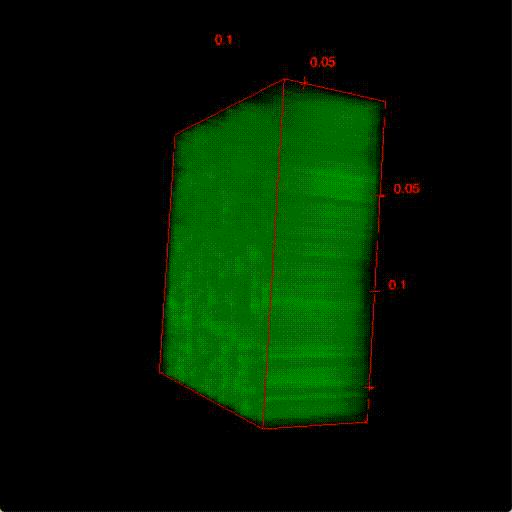


**Supplementary Video 2.** Animation of anti-collagen MNP-dosed collagen I hydrogel aligned by application of magnetic field. Generated in Imaris software using z-stack images obtained through confocal microscopy. 360^o^ rotation shown.


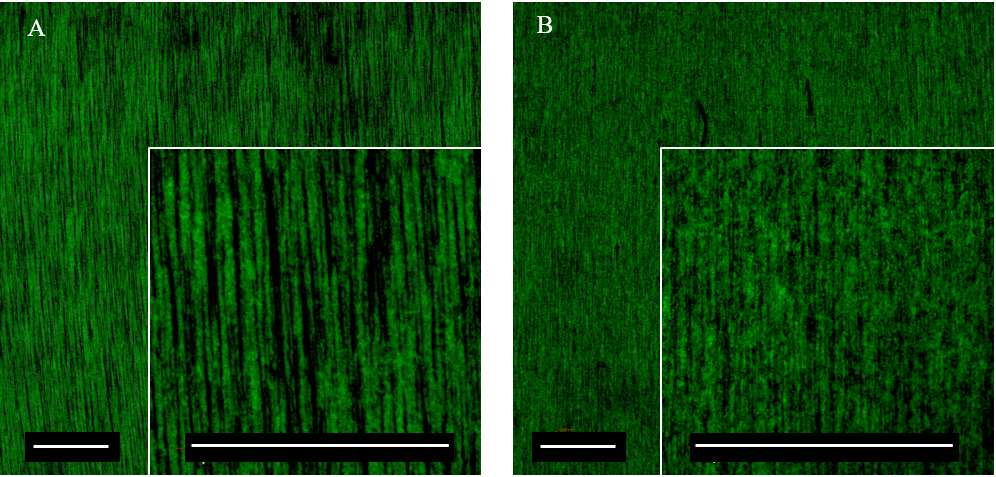


**Supplementary Figure 2.** Confocal images of 3 mg/mL collagen I hydrogel dosed with 1 μg/μL anti-collagen MNPs acquired 21-days post magnetic stimulation. Anisotropic fiber-like structure alignment was still present post 21-days. Scale bar = 0.5 mm, magnification = 4× and 20×, green = collagen I, n = 3.

**Supplementary Figure 3.** Cell viability of human adipose stem cells after 3 days incubation in 1 μg/μL MNP-dosed 3 mg/mL collagen hydrogels. Cell viability is normalized to the positive control; collagen hydrogels with no MNP dosing. The control group had an average viability of 97.83% suggesting that MNPs have no cytotoxic effect, n = 3.

**
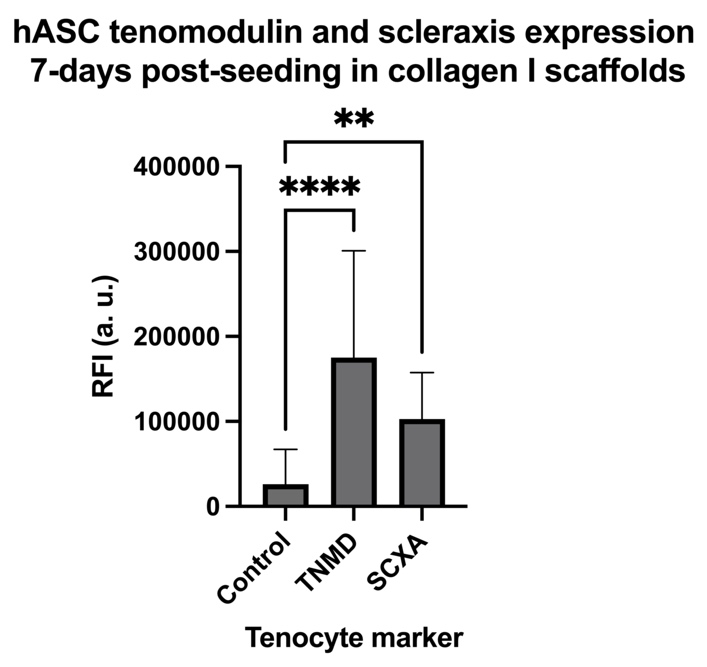
**

**Supplementary Figure 4.** Average relative fluorescent intensity (RFI) of tenomodulin (TNMD) and scleraxis (SCXA) proteins, compared to control. Confocal microscope acquisition settings were standardized for all images. Fluorescent intensity was quantified using the analyze function of FIJI (v2.3.0). There is a significant difference between the control and experimental groups, indicating that the fluorescent output is indeed related to abundance of immunofluorescently tagged proteins, and background noise and/or non-specific staining is insignificant. Measurements were taken from three randomly selected regions of interest (100 x 100 pixels) per image. The average and standard deviation is plotted. A standard one-way ANOVA was used to determine significance. hASC = human adipose-derived stem cells, n = 3.
